# Supplementary material for: Membrane potential and feedback dynamics regulate CatSper-mediated progesterone signaling in human sperm
Source: bioRxiv. 2025 Sep 17:2025.09.14.675619. Preprint. [Version 1] doi: 10.1101/2025.09.14.675619 (PMC12458478; doi:10.1101/2025.09.14.675619)
Supplement: 1 [file NIHPP2025.09.14.675619V1-supplement-1.pdf]

bioRxiv preprint doi: https://doi.org/10.1101/2025.09.14.675619; this version posted September 17, 2025. The copyright holder for this preprint (which was not certified by peer review) is the author/funder, who has granted bioRxiv a license to display the preprint in perpetuity. It is made available under aCC-BY 4.0 International license.

Supplementary Materials

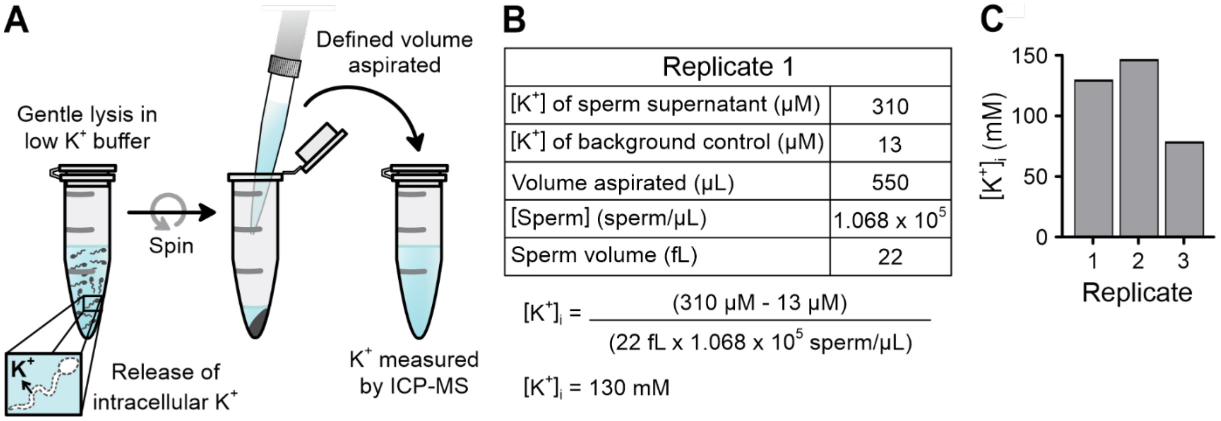

Supplementary Figure 1. Determination of the intracellular K^+ concentration in human sperm. (A) Schematic depicting how the intracellular K^+ concentration ($[K^+]_i$) was determined. Intracellular K^+ from a dense sperm suspension of known concentration was released by permeabilizing the plasma membrane with a hypotonic, low K^+ buffer containing 0.001% digitonin. This release elevated the K^+ concentration in the medium. After centrifugation, a defined volume of the supernatant (550 µl) was aspirated, and the K^+ concentration was determined by inductively coupled mass spectrometry (ICP-MS). **(B)** Results and calculation for one representative replicate. The protocol outlined in (A) was performed in parallel for an equivalent volume of sperm-free medium; this background control accounts for the K^+ in the medium prior to K^+ release from sperm. **(C)** Graph showing the $[K^+]_i$ result for each replicate. The mean $[K^+]_i$ was 120 ± 40 mM (n = 3).

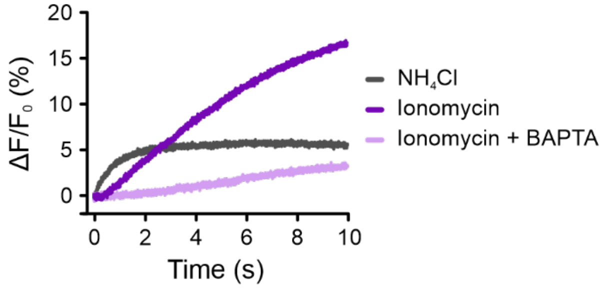

Supplementary Figure 2. Ionomycin-induced changes in intracellular pH. Increase in intracellular pH (pH_i) induced by mixing human sperm with ionomycin (10 µM) or NH_4Cl (30 mM), reported by pHrodo. The pH_i response was attenuated when ionomycin was co-delivered with the extracellular Ca^{2+} chelator BAPTA (3.2 mM), adjusting $[Ca^{2+}]_o$ to < 100 nM. This reinforces that the pH_i response arises from the Ca^{2+}/H^+ exchange by ionomycin.

bioRxiv preprint doi: https://doi.org/10.1101/2025.09.14.675619; this version posted September 17, 2025. The copyright holder for this preprint (which was not certified by peer review) is the author/funder, who has granted bioRxiv a license to display the preprint in perpetuity. It is made available under aCC-BY 4.0 International license.

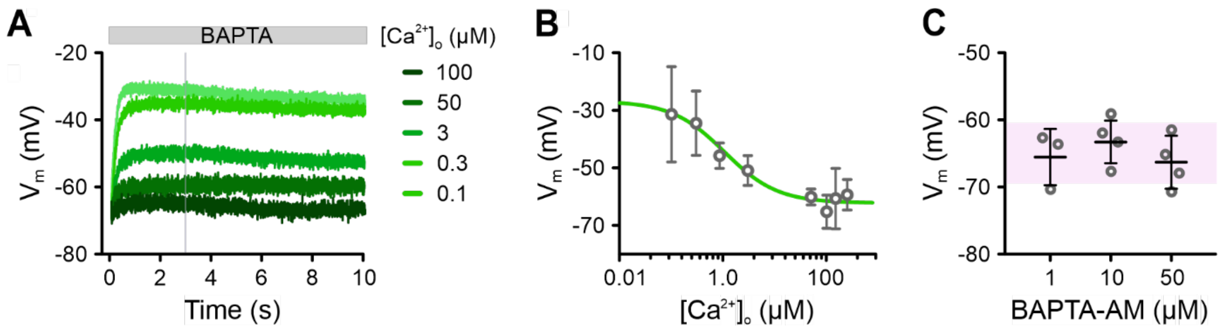

Supplementary Figure 3. The action of low extracellular Ca^{2+} and buffering of intracellular Ca^{2+} by BAPTA on the membrane potential of human sperm. (A) Average (n ≥ 3) changes in V_m of non-capacitated VF2.1.Cl-loaded sperm induced by mixing with varying concentrations of BAPTA, reducing extracellular Ca^{2+} ($[Ca^{2+}]_o$) from 1.6 mM to the values indicated. Error bars were omitted for clarity. Fluorescence was converted into millivolts by accompanying null-point calibrations (not shown). **(B)** Characterization of the relationship between the V_m response, averaged over the grey window in (A), and the final $[Ca^{2+}]_o$. The V_m response was attenuated at the half-maximal 'inhibitory' concentration (IC_{50}) of 1.1 μM $[Ca^{2+}]_o$, which aligns with the IC_{50} values for the inhibition of monovalent CatSper currents by extracellular Ca^{2+} (Lishko et al., 2011; Smith et al., 2015). **(C)** Mean (± SD) V_{rest} of non-capacitated VF2.1.Cl-loaded sperm after loading for 60 min with either 1 μM, 10 μM, or 50 μM BAPTA-AM. The shaded region shows the V_{rest} (± SD) of control, unloaded sperm from Figure 2F (-65 ± 5 mV; n = 110).

bioRxiv preprint doi: https://doi.org/10.1101/2025.09.14.675619; this version posted September 17, 2025. The copyright holder for this preprint (which was not certified by peer review) is the author/funder, who has granted bioRxiv a license to display the preprint in perpetuity. It is made available under aCC-BY 4.0 International license.

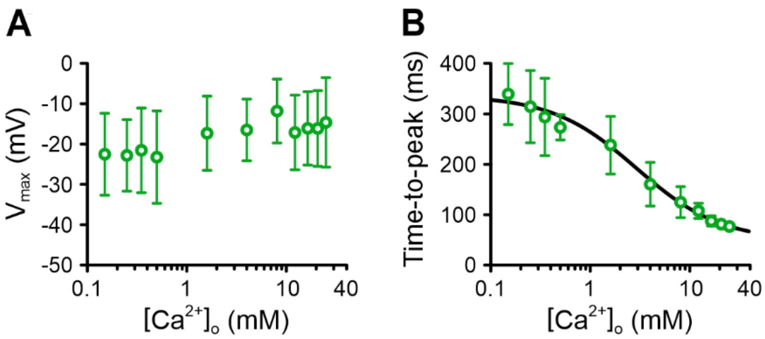

Supplementary Figure 4. The action of extracellular Ca^{2+} on progesterone-induced V_m responses in human sperm. (A, B) The mean (± SD) **(A)** V_{max} and **(B)** time-to-peak (peak = V_{max}) of progesterone-induced V_m responses at varying $[Ca^{2+}]_o$, shown in Figure 7A and C (n ≥ 4). The time-to-peak was half-maximal (EC_{50}) at 3 mM $[Ca^{2+}]_o$.

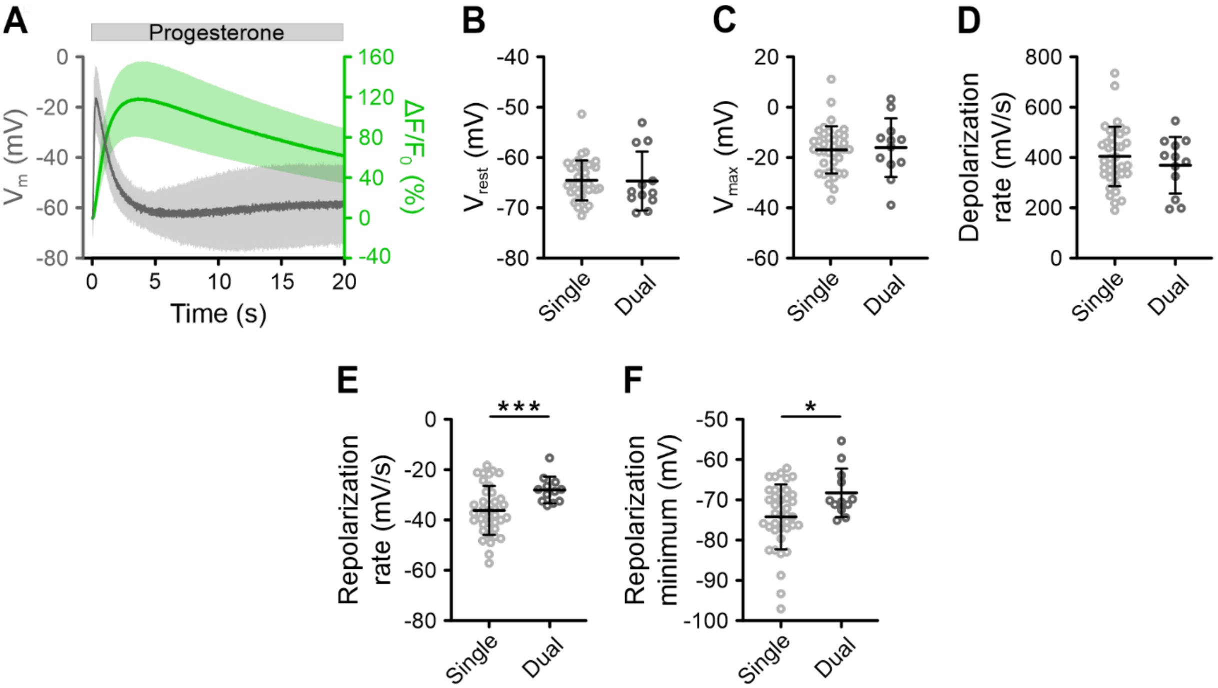

Supplementary Figure 5. Properties of the progesterone-evoked V_m signal recorded individually compared to simultaneously with the Ca^{2+} signal. (A) Progesterone-evoked V_m and Ca^{2+} signals (mean ± SD; n = 12) in non-capacitated human sperm loaded with VF2.1.Cl and Calbryte 630, recorded simultaneously with FAST[M] at 1.6 mM $[Ca^{2+}]_o$. **(B–F)** Mean (± SD) **(B)** V_{rest}, **(C)** V_{max}, **(D)** depolarization rate, **(E)** repolarization rate, and **(F)** repolarization minimum determined for progesterone-evoked V_m signals recorded from VF2.1.Cl-loaded, non-capacitated sperm either with (n = 12) or without (n = 38) loading with the Ca^{2+} indicator Calbryte 630, referred to in the figure as dual and single recording, respectively. Sparse loading with Calbryte 630 resulted in some buffering of $[Ca^{2+}]_i$, resulting in mild attenuations in the Ca^{2+}-dependent repolarization phase. * $p < 0.05$, *** $p < 0.001$.

bioRxiv preprint doi: https://doi.org/10.1101/2025.09.14.675619; this version posted September 17, 2025. The copyright holder for this preprint (which was not certified by peer review) is the author/funder, who has granted bioRxiv a license to display the preprint in perpetuity. It is made available under aCC-BY 4.0 International license.

Supplementary Table 1. Literature review of previous determinations of non-capacitated and capacitated human sperm V_{rest}.

V_{rest} (mV)		Methodology	Publications
non-capacitated	capacitated		
-69 ± 2<br>mean ± SD; n = 6	-36 ± 3#<br>mean ± SD; n = 4	Radioactivity assay	Calzada *et al*., 1988
-75 ± 6<br>mean ± SD; n = 4	not determined	Radioactivity assay	Calzada & Tellez, 1997
-40 ± 16<br>mean ± SD; n = 7	not determined	Spectrofluorimetry with 500 nM $DiSC_3(5)$	Linares-Hernandez *et al*., 1998
not determined	-58 ± 2<br>mean ± SEM; n = 12	Spectrofluorimetry with 250 nM $DiSC_3(5)$	Patrat *et al*., 2002
not determined	-36.5 ± 3.3<br>(mean ± SEM); n = 33	Patch-clamp under quasi-physiological gradients	Mansell *et al*., 2014
-17.7 ± 1.8<br>mean ± SEM; n = 10	-22.7 ± 2<br>mean ± SEM; n = 16	Patch-clamp under quasi-physiological gradients	Brown *et al*., 2016
-63.5<br>n = 1	not determined	Spectrofluorimetry with 1 µM $DiSC_3(5)$	Baro Graf *et al*., 2019
-63.1<br>n = 1	-74.5<br>n = 1	Flow cytometry with 50 nM $DiSC_3(5)$	
-37.7 ± 9.9<br>mean ± SD; n = 29	-57.8 ± 12.9<br>mean ± SD; n = 29	Spectrofluorimetry with 1 µM $DiSC_3(5)$	Baro Graf *et al*., 2020
-55 ± 14#<br>mean ± SD; n = 18	-38 ± 5#<br>mean ± SD; n = 18		
-39.7 ± 8#<br>mean ± SD; n = 13	-38.9 ± 10#<br>mean ± SD; n = 13		
-35.7 ± 2.8<br>mean ± SEM; n = 13	-45.2 ± 3.2<br>mean ± SEM; n = 13	Flow cytometry with 5 nM $DiSC_3(5)$	Molina *et al*., 2020
-67 ± 4<br>mean ± SEM; n = 9	-70 ± 3<br>mean ± SEM; n = 9	Flow cytometry with 25 nM $DiSC_3(5)$	Matamoros-Volante *et al*., 2020
-60 ± 3<br>mean ± SEM; n = 6	-71 ± 3<br>mean ± SEM; n = 6		

Publications are listed chronologically and colour-coded based on method. Green: radioactivity assay, yellow: spectrofluorimetry with $DiSC_3(5)$, orange: flow cytometry with $DiSC_3(5)$, and blue: sperm patch-clamp under quasi-physiological conditions.

# Indicates that values were estimated from figures as they were not explicitly given.

bioRxiv preprint doi: https://doi.org/10.1101/2025.09.14.675619; this version posted September 17, 2025. The copyright holder for this preprint (which was not certified by peer review) is the author/funder, who has granted bioRxiv a license to display the preprint in perpetuity. It is made available under aCC-BY 4.0 International license.

Supplementary Table 2. Literature review of non-capacitated and capacitated mouse sperm V_{rest}.

V_{rest} (mV)		Methodology	Publication
**non-capacitated**	**capacitated**		
-42 ± 8.8<br>mean ± SD; n = 16	not determined	Spectrofluorimetry with 1 μM $DiSC_3(5)$	Espinosa & Darszon, 1995
-38 ± 4<br>mean ± SD; n = 3	-55 ± 2<br>mean ±SD; n = 4	Spectrofluorimetry with 250 nM $DiSC_3(5)$	Zeng *et al.*, 1995
-33 ± 4<br>mean ± SD; n = 3	-49 ± 3<br>mean ± SD; n = 3	Spectrofluorimetery with 500 nM $DiSBAC_2(3)$	
-25.7 ± 6.6<br>mean ± SD; n = 75	-63.4 ± 17.1<br>mean ± SD; n = 61	Single cell fluorimetry with 1 μM di-8-ANEPPS	Arnoult *et al.*, 1999
-52 ± 6<br>mean ± SD; n = 10	-66 ± 9 mV<br>mean ± SD; n = 10	Spectrofluorimetry with 1 μM $DiSC_3(5)$	Munoz-Garay *et al.*, 2001
-36 ± 2#<br>mean ± SEM; n = 3	-67 ± 2#<br>mean ± SEM; n = 3	Spectrofluorimetry with 1 μM $DiSC_3(5)$	Demarco *et al.*, 2003
-47 ± 3.3 mV<br>mean ± SEM; n = 4	-62.2 ± 2.9<br>mean ± SEM; n = 4	Spectrofluorimetry with 1 μM $DiSC_3(5)$	Santi *et al.*, 2010
-45.59 ± 2.57<br>mean ± SEM; n = 11	-63.88 ± 2.92<br>mean ± SEM; n = 11	Spectrofluorimetry with 1 μM $DiSC_3(5)$	Chavez *et al.*, 2013
-42.6<br>n = 1	-62.4<br>n = 1	Spectrofluorimetry with 1 μM $DiSC_3(5)$	Escoffier *et al.*, 2015
not determined	-59.4 ± 5.3<br>mean ± SEM; n = ?	Flow cytometry with 5 nM $DiSC_3(5)$	Molina *et al.*, 2020

Publications are listed chronologically and colour-coded according to method: Yellow: spectrofluorimetry with $DiSC_3(5)$, orange: flow cytometry with $DiSC_3(5)$, pink: single-cell fluorimetry with di-8-ANEPPS, grey: spectrofluorimetry with $DiSBAC_2(3)$. Although mouse sperm V_m has been extensively studied by sperm patch-clamp, these publications were excluded from the table as recordings were not performed under physiological conditions at pH 6.5.

# Indicates that values were estimated from figures as they were not explicitly given.

? Indicates that n values could not be identified.
